# Cellular and Humoral Immune Responses and Breakthrough Infections After Two Doses of BNT162b Vaccine in Healthcare Workers (HW) 180 Days After the Second Vaccine Dose

**DOI:** 10.3389/fpubh.2022.847384

**Published:** 2022-03-31

**Authors:** Alessandra Mangia, Nicola Serra, Giovanna Cocomazzi, Vincenzo Giambra, Stefano Antinucci, Alberto Maiorana, Francesco Giuliani, Emanuele Montomoli, Paolo Cantaloni, Alessandro Manenti, Valeria Piazzolla

**Affiliations:** ^1^Liver Unit, Fondazione IRCCS “Casa Sollievo della Sofferenza”, San Giovanni Rotondo, Italy; ^2^Department of Public Health, University “Federico II”, Naples, Italy; ^3^Institute for Stem Cell Biology, Regenerative Medicine and Innovative Therapies (ISBReMIT), San Giovanni Rotondo, Italy; ^4^Allergy Diagnostic Section Euroimmun, Italy Fondazione “Casa Sollievo della Sofferenza”, San Giovanni Rotondo, Italy; ^5^GSSL Unit, Fondazione “Casa Sollievo della Sofferenza”, San Giovanni Rotondo, Italy; ^6^ICT Innovation and Research Unit, Fondazione IRCCS “Casa Sollievo della Sofferenza”, San Giovanni Rotondo, Italy; ^7^Department of Molecular and Developmental Medicine, University of Siena, Siena, Italy; ^8^VisMederi Srl, Siena, Italy

**Keywords:** SARS-CoV-2, mRNA vaccines, humoral response, IFN-γ, healthcare workers

## Abstract

**Background:**

Immunity and clinical protection induced by mRNA vaccines against SARS-CoV-2 have been shown to decline overtime. To gather information on the immunity profile deemed sufficient in protecting against hospitalization, we tested IgG levels, interferon-gamma (IFN-γ) secretion, and neutralizing antibodies 180 days (d180) after the second shot of BNT162b vaccine, in HW.

**Methods:**

A total of 392 subjects were enrolled. All received BioNTech/Pfizer from February 2020 to April 2021. The vaccine-specific humoral response was quantitatively determined by testing for IgG anti-S1 domain of SARS-CoV-spike protein. Live virus microneutralization (MN) was evaluated by an assay performing incubation of serial 2-fold dilution of human serum samples, starting from 1:10 to 1:5120, with an equal volume of Wuhan strain and Delta VOC viral solution and assessing the presence/absence of a cytopathic effect. SARS-CoV-2-spike protein-specific T-cell response was determined by a commercial IFN-γ release assay.

**Results:**

In 352 individuals, at d180, IgG levels decreased substantially but no results below the assay's positivity threshold were observed. Overall, 22 naive (8.1%) had values above the highest threshold. Among COVID-naive, the impact of age, which was observed at earlier stages, disappeared at d180, while it remained significant for 81 who had experienced a previous infection. Following the predictive model of protection by Khoury, we transformed the neutralizing titers in IU/ml and used a 54 IU/ml threshold to identify subjects with 50% protective immunity. Overall, live virus MN showed almost all subjects with previous exposure to SARS-CoV-2 neutralized the virus as compared to 33% of naive double-dosed subjects (*p* < 0.0001). All previously exposed subjects had strong IFN-γ secretion (>200 mIU/ml); among 271 naive, 7 (2.58%) and 17 (6.27%) subjects did not show borderline or strong secretion, respectively.

**Conclusions:**

In naive subjects, low IgG titers are relatively long-lasting. Only a third of naive subjects maintain neutralizing responses. After specific stimulation, a very limited number of naive were unable to produce IFN-γ. The results attained in the small group of subjects with breakthrough infection suggest that simultaneous neutralizing antibody titers <20, binding antibody levels/ml <200, and IFN-γ <1,000 mIU/ml in subjects older than 58 may identify at-risk groups.

## Introduction

Several studies on the durability of humoral response in subjects recovered from SARS-CoV-2 infection showed that both binding and neutralizing antibody levels decrease only modestly at month 8 after the infection (1, 2). This evidence initially suggested that vaccinated persons and previously infected would experience a low number of breakthrough infections. However, the durability of immunity has been called into question by the mounting evidence of reinfections after natural recovery (3). Moreover, a progressive decline in humoral immune response has been shown after vaccination (4). In our experience, in a cohort of healthcare workers, this decline was shown to start from d90 after the first shot (5). These results were in agreement with larger cohort studies (4) and suggest that after vaccination or infection, several mechanisms of immunity exist both at the antibody level and at the level of cellular immunity.

Moderna and Pfizer vaccines using a mutated sequence of the receptor-binding domain (RDB) that contains two consecutive prolines, lysine 986, and valine 987 (6) have been associated with high protection rates (7). Accumulating evidence demonstrates that the two doses of the BNT162b vaccine elicit either high IgG or neutralizing antibody responses (8, 9). Neutralizing antibodies were shown to correlate with protection and may be used to assess effective vaccine-induced humoral response (10) However, there is scarce applicability of neutralizing assays in the routine practice as neutralizing tests are complex, time-consuming, and not always comparable across assays (11). In addition, a time-dependent neutralizing activity regression relationship with IgG levels has been demonstrated (4).

It has recently been shown that fully vaccinated people remain at the risk for SARS-CoV-2 infections and Pfizer's CEO announced in October 2021, the need for a booster within 12 months of the first dose (12–14). In a recent study from Israel, involving participants 60 years old, 5 months after two doses of BioNTech/Pfizer vaccine, rates of infection and severe illness were lower among those who received a booster injection as compared to participants who did not (15).

Evidence suggests that humoral response alone may not offer sufficient protection against either infection or disease, and SARS-CoV-2-specific cellular immunity may be more stable and longer-lasting than humoral immunity (1). It has been, therefore, hypothesized, based on experimental models, that CD4+ and CD8+ T-cells and production of IFN-γ play an important role in vaccination immune response (16).

We analyzed – by age, gender, and previous SARS-CoV-2 infection history –the binding and neutralizing antibody response induced by the BioNTech/Pfizer vaccine 180 days after the second vaccine shot in our cohort of almost 400 healthcare workers longitudinally followed up to 180 days after the second dose of BioNTech/Pfizer. The subjects' early humoral response had been previously reported to decline 90 days after the first vaccine dose (5). Spike-specific T-cell-mediated reactivity using an IFN-γ release assay, with the aim to gather information about cellular immune response, was also evaluated.

## Methods

Our analysis was based on the medical data from the multicenter longitudinal study (Covidiagnostix, funded by the Italian Ministry of Health) to investigate the antibody response in Healthcare workers vaccinated with BioNTech/Pfizer starting from February 11, 2020, and ending on April 11, 2021. All the subjects received two vaccine injections 21 days apart. The planned testing time for binding antibodies was day 0 (d0) (before the first dose), day 7 (d7), day 21 (d21), day 31 (d31) after the first shot, and day 90 (d90) 60 days after the second shot, day 180 (d180) days after the second shot corresponding to 210 days after the first shot, respectively.

We excluded the participants who do not have the complete set of blood sample collection. Blood samples were collected into clot activator BD vacutainer tubes (Becton Dickinson, Franklin Lakes, NJ, USA). The margin of sampling window for each time-point was of 2 days.

### Antibody Evaluation

The vaccine-specific humoral immune response was quantitatively determined by testing for antiS1 and SARS-CoV-spike protein (EUROIMMUN, anti-SARS-CoV-2 QuantiVac enzyme-linked immunosorbent assay) with a positive cut-off of at least 3.2 Binding Arbitrary Unit (BAU) ml. This assay was designed to evaluate vaccine response and calibrated against WHO standards in order to provide results in BAU (17). The cut-off for positivity was 35.2 BAU, low quantitation limit 3.2 BAU/ml at 1:101 dilution, and range (3.2–384.0 BAU/ml). Results 25.6 but <35.2 were considered borderline (18). Specificity and sensitivity (>10 days after diagnosis) are 99.8 and 90.3%, respectively, when the manufacturer's suggested cut-off of 35.2 BAU/ml is used. A solution used for diluting samples above 348 U/ml was included in the measurement kits.

The SARS-CoV-2 spike protein-specific T-cell response was determined by a commercial, standardized interferon-gamma (IFN-γ) release assay (IGRA) using the EUROIMMUN SARS-CoV-2 IGRA stimulation tube set (product No. ET 2606-3003) and EUROIMMUN IFN-γ ELISA (product No. EQ 6841-960). The specific T-cell response was quantified according to the manufacturer's instructions and values >100 mIU/ml were interpreted as low positive, >200 mIU/ml as positive (19).

### Cell Culture

VERO E6 C1008 cells (CRL-1586) were cultured in Dulbecco's Modified Eagle's Medium (DMEM), High Glucose (Euroclone), supplemented with 2 mM L-glutamine (Lonza), 100 units/ml Penicillin–Streptomycin mixture (Lonza), and 10% fetal bovine serum (FBS) (Euroclone), in 37°C and 5% CO2 humidified incubator. Adherent sub confluent cell monolayers of VERO E6 were prepared in DMEM high glucose containing 2% FBS in 96 well plates for virus titration and neutralization tests.

### Micro-Neutralization Experiments

The micro-neutralization (MN) assay was performed as previously reported (20, 21). Briefly, serial 2-fold dilution of human serum samples, starting from 1:10 to 1: 5120, were incubated with an equal volume of SARS-CoV-2 (Wuhan Strain and Delta VOC) viral solution containing 25 tissue culture infective dose 50% (TCID50) for 1 h at RT (21). After incubation, 100 μl of the serum–virus mixture was transferred to a 96-well plate containing an 80% sub-confluent Vero E6 cell monolayer. The plates were incubated for 3 days (Wuhan strain) and 4 days (Delta strain) at 37°C and 5% CO_2_. At the end of incubation, the presence/absence of cytopathic effect (CPE) was assessed by means of an inverted optical microscope. A CPE higher than 50% was indicative of infection. The MN titer was expressed as the reciprocal of the highest serum dilution showing protection from viral infection and CPE. The titer of 10 was considered as the lower limit of quantitation (LLOQ) and a titer equal to 5 was considered as negative. All experiments with live SARS-CoV-2 viruses were performed inside the Biosecurity Level 3 laboratories of VisMederi Srl. Standardization of neutralizing titers was made following the guidelines of the NIBSC 20/136 document^1^.

### COVID-19 Diagnostic Data

As part of preventive medicine practice, healthcare workers were subjected to routine RT-PCR swab testing using a Real-Time Reverse transcription PCR kit on a Roche Cobas Z480 thermocycler (Roche Diagnostic, Basel, Switzerland). RNA purification was performed using Roche Magna pure system (Roche Diagnostic, Basel, Switzerland). Both the results of the swab test and the clinical information collected in a dedicated questionnaire were used to confirm the previous SARS-CoV-2 infection and were compared to the results of the COVID-19 Regional Registry.

### Ethics Approval

All healthcare workers provided written consent in accordance with local review board requirements. Laboratory investigations and available clinical data were collected and analyzed according to the protocol COVIDIAGNOSTIX approved by the EC review board at our institution and funded by the Ministry of Health of Italy, “Bando Ricerca COVID-19,” project number: COVID-2020-12371619; project title: COVIDIAGNOSTIX—Health Technology Assessment in COVID serological diagnostics.

### Statistical Analysis

Data were presented as numbers and percentages for categorical variables. Continuous variables were expressed as mean ± *SD* or median with interquartile range (*IQR*). Test for Normal distribution was performed by Shapiro–Wilkson test. The *T*-test was used to compare the mean of unpaired samples. When the distribution of samples was not normal, a *T*-test with logarithmic transformation was performed. Alternative non-parametric tests such as Mann–Whitney test were used when distribution was not normal. Differences between groups were analyzed using the chi-square test or Fisher's exact test for categorical variables.

Linear regression was used to describe the relationship between two variables and to predict one variable from another. In a scatter diagram with a regression line, the relation between two variables was presented graphically, and the linear correlation coefficient and *p*-value were reported.

Tests with *p-value (p)* < 0.05 were considered significant. The statistical analysis was performed by Matlab statistical toolbox version 2008 (MathWorks, Natick, MA, USA) for Windows at 32 bit.

Logistic regression was used to find the best fitting model to describe the relationship between the dichotomous characteristic of interest (dependent variable) and a set of independent variables.

## Results

### Serological Evaluation by the Previous History of SARS-CoV-2 Infection at day 180 After the Second Dose

Of 392 enrolled subjects, 352 were analyzed, as 40 (10.2%) had to be excluded because they did not complete the planned sample collection. The mean age was 47.7 years ± 11.8. Of the total participants, 57.2% were female; 271 had no experience of the previous infection and were defined as naive. Subjects infected before or immediately after the first vaccine dose (*n* = 81) were classified as experienced.

Of 271 naive, the female prevalence was 58.3%, and the mean age was 47.55 years ± 11.85. The mean values of IgG antibodies were 212.93 ± 182.98 BAU/ml (Table 1). None had results below the 35.2 BAU/ml positivity assay threshold. Overall, 22 individuals (8.1%) had antibody values above the highest threshold. Their mean values were 630.50 ± 361.46 BAU/ml. No difference was observed by gender.

**Table 1:** Baseline characteristics, antibody levels, neutralizing antibody titers, and IFN-γ concentration of vaccinated subjects.

	**Prior COVID-19 experience**		
	**Yes (*n* = 81)**	**No (*n* = 271)**	***p* value**
Age, mean (SD), years Median (IQR)	49.71 (12.32) 51 (40.75–59.25)	47.55 (11.85) 47 (39.0–57.0)	0.20
Sex: Male Female	38 (46.9) 43 (53.1)	113 (41.7) 158 (58.3)	0.41
Baseline SARS-CoV-2-IgG No (%)	79 (97.31)	0	*p* < 0.0001
Day 180 SARS-CoV-2-IgG, No (%)	81(100)	271 (100)	*p* = 1
Day 180 SARS-CoV2-IgG level Mean, (SD) BAU/ml Median (IQR)	418.81 ± 415.01 248.96 (140.48–610.0)	212.93 ± 182.98 179.79 (90.0–287.19)	*p* < 0.0001
Day 180^*^ SARS-CoV2-IgG level >384 BAU/ml Mean, (SD) BAU/ml Median (IQR)	778.04 ± 40.15 630.41 (548.32–895.72)	630.50 ±361.46 489.93 (398.31–666.08)	*p* = 0.092
Day 180 Neutralizing antibody >10, No (%)	81 (100)	178 (65.89)	<0.0001
Day 180 Neutralizing antibody Mean (SD) Median (IQR)	419.08 ± 430.75 231.52 (138.46-612.16)	229.27 ± 213.92 200 (90.0–310–72)	*p* = 0.0009
Day 180^**^ Neutralizing antibody >320 Mean, SD Median (IQR)	740.24 ± 588.37 663.36 (209.04–921.54)	246.09 ± 65.17 246.09 (200.0–292.17)	*p* = 0.32
Day 180 IFN-γ No (%) >100 mIU/ml	81 (100)	267 (98.52)	0.58
Day 180 IFN-γ No (%) >200 mIU/ml	81 (100)	254 (93.72)	0.0161
Day 180 IFN-γ >100 mIU/ml Mean (SD) Median (IQR)	2,299.97 ± 491.25 2,499.0 (2,400.0–2,500.0)	1,201.24 ± 846.24 926.0 (463.0–2,272.0)	*p* < 0.0001

* *IgG Mean values for subjects with results above the highest threshold of the assay*;

** * Mean titers of neutralizing antibodies among subjects with titers associated with strong neutralizing capacity*.

**Figure 1:**
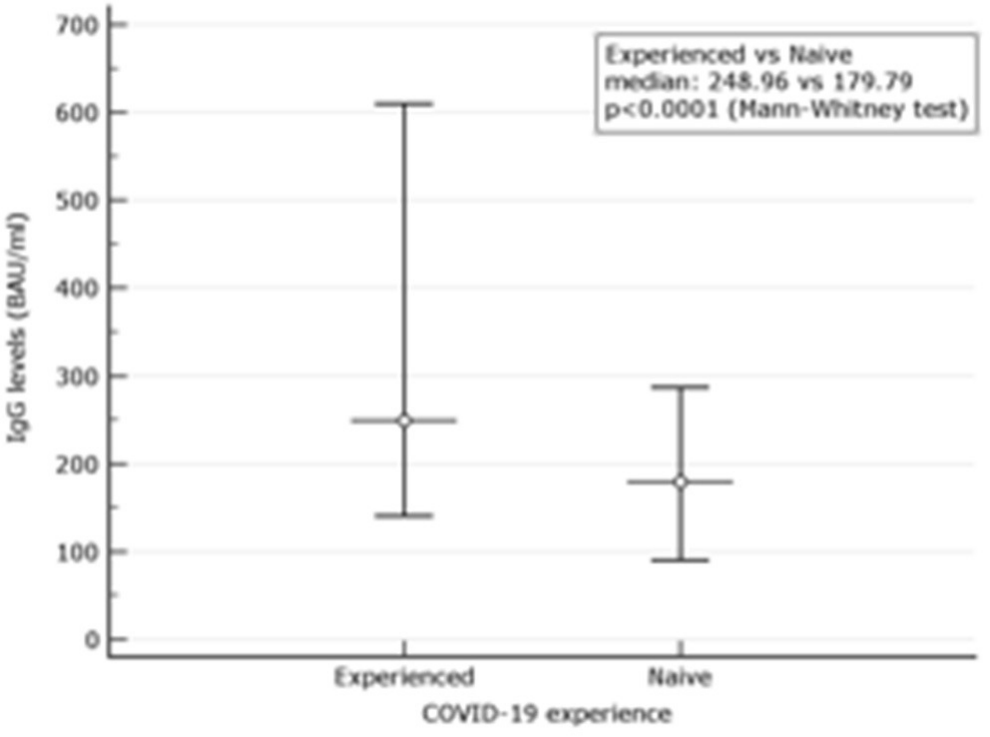
Comparison between IgG levels in naive and experienced. Mean and Interquartile ranges (*IQR*) are reported (*p* < 0.0001).

Among 81 experienced, the female was 53.1%. The mean age was 49.71 ± 12.32. At d180 after the second dose (210 days after the first vaccination), the mean values were 418.81 BAU/ml ± 415.01. None had results below the assay's threshold. Overall, 41.03% had results above the 384.0 BAU/ml (Table 1). Their mean values were 778.04 ± 40.15 BAU/ml. Values for men and women were not different regardless of the threshold used. Comparison between IgG levels in naive and experienced is depicted in a graph (Figure 1).

**Figure 2:**
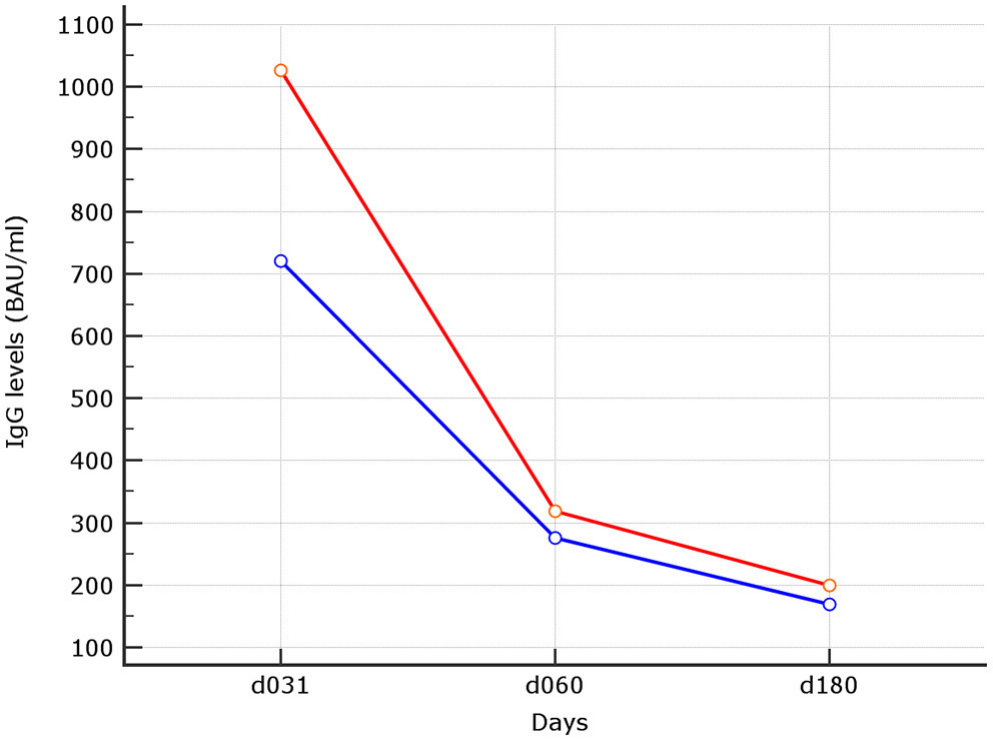
Trend analysis of IgG levels at the different time points. In red median of IgG levels in subjects with median age ≤ 47 years. In blue median of IgG levels in subjects older than 47 years, linear trend was statistically significant for both (*p* < 0.0001).

The impact of age on binding antibody levels was then investigated (Table 2). Within the naive group, stratification of binding antibody levels by median age of 47 years revealed no difference. When subjects older than 47 years were compared to the younger patients, median levels of 169.84 (90.0–268.87) BAU/ml *vs*. 200.0 (95.63–298.87) BAU/ml (*p* = 0.40) were observed. At variance, within the experienced group, older had higher median age than younger 412.82 (165.44–642.93) *vs*. 211.36 (126.40–310.00) (*p* = 0.0043). This inverse relationship with the age within the experienced group was also observed although at a not significant level at d90, 60 days after the second shot (*p* = 0.087). At earlier time points, as reported in our previous experience (5), the difference between higher median IgG levels in younger *vs*. older was significant also within the naive group (median age of younger of 1026.0 (489.01 *vs*. 1690.01) *vs*. 720.12 (479.35–1251.02) (*p* = 0.022). Trend analysis of the three different time points IgG levels using median was performed (*p* < 0.0001 for both younger and older than 47 years) (Figure 2).

**Table 2:** Comparison of IgG levels in subjects previously infected or naive by age younger or older than 47 years.

IgG levels (age ≤ 47) Mean±SD Median (IQR)	224.58 ± 198.12 200.0(95.63;298.87)	274.11 ± 231.78 211.36 (126.40;310.0)	0.32
IgG levels (age >47) Mean±SD Median (IQR)	200.75 ± 165.55 169.84(90.0;268.87)	530.62 ± 487.68 412.82 (165.44;642.93)	<0.0001

*SD, standard deviation; IQR, Interquartile range; Median and IQR were used for data with no normal distribution*.

### Neutralizing Antibodies Results

**Figure 3:**
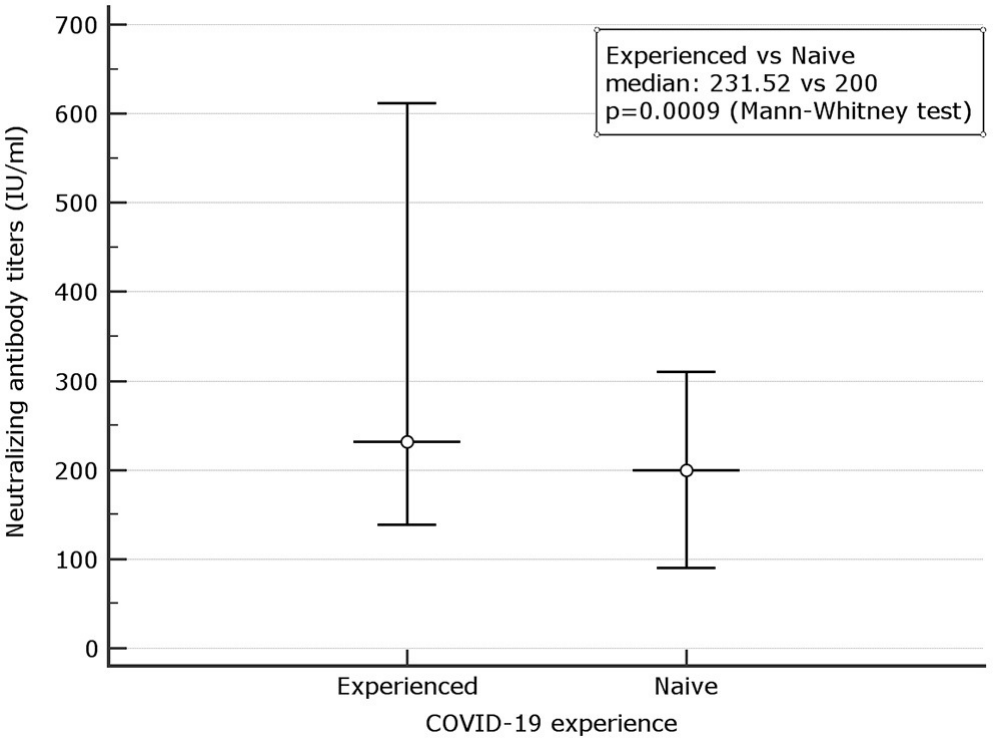
Comparison between microneutralization results in naive and experienced. Mean and Interquartile ranges (*IQR*) are reported (*p* = 0.0009).

When the neutralizing titers were analyzed, 100% of previously infected patients and 178 (65.89%) of naive showed a titer of ≥10 (LLoQ). Individuals with titers associated with stronger neutralizing capacity associated to a dilution > 320 were 2 (0.73%) among naive and 25 (31.2%) among 80 experienced (*p* < 0.0001). Median neutralizing titers of 200 (90.0–310.72) were observed among 271 naive. The corresponding value among experienced was 231.52 (138.46–612.16) (Figure 3). When only subjects with strong neutralizing titers (>320) were analyzed, the median titers were 246.09 (200.0–292.17) for naive and 663.36 (209.04–921.54) for experienced. Following the predictive model of protection suggested by Khoury et al. (22) and using the standard IU/ml results suggested by WHO as a reference to normalize the different neutralizing testing^1^, we transformed the neutralizing titers in IU/ml and used a 54 IU/ml threshold to identify subjects with 50% protective humoral immunity. Overall, 32.78% of naive and 91.89% of previously infected (*p* < 0.0001) showed protective neutralizing activity.

### Correlation Between IgG and Neutralizing Antibodies

**Figure 4:**
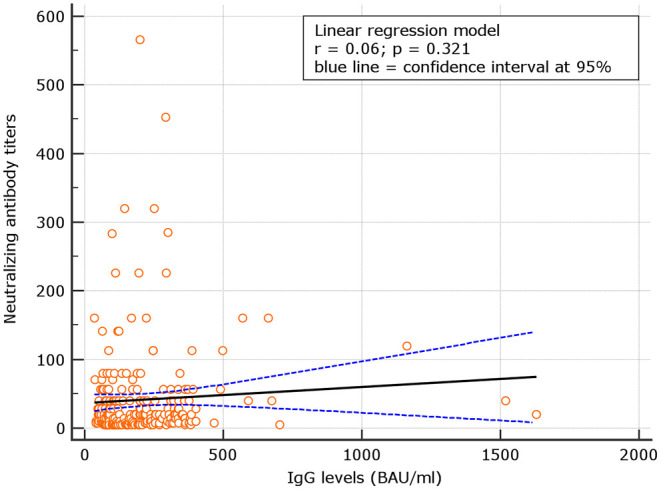
Correlation between neutralizing antibody titers and IgG levels among naive.

**Figure 5:**
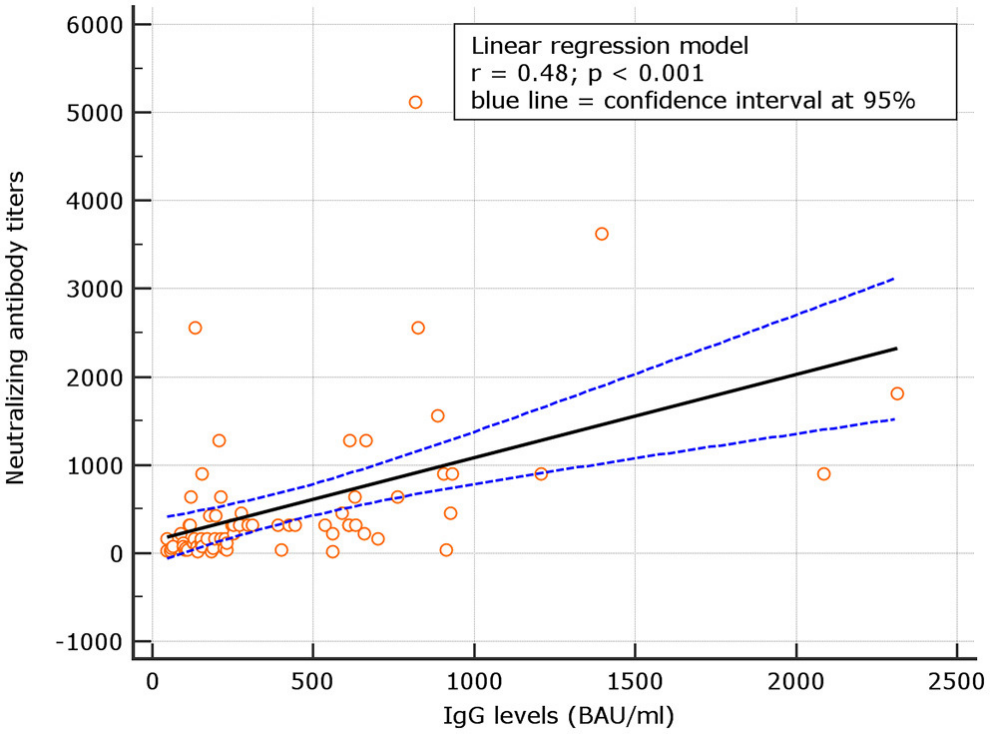
Correlation between neutralizing antibody titers and IgG levels among experienced.

No correlation was observed between neutralizing antibody titers and IgG levels for naive (*r* = 0.06; *p* = 0.321), at d180. At variance, for experienced, the correlation was significant (*p* = 0.48; *p* < 0.001) (Figures 4, 5). Despite the analysis of neutralizing antibody, IU/ml ≥54 conversions, we failed to observe correlation with binding antibody.

### IFN-γ Results

The spike-specific T-cell response was assessed by semi-quantitative analysis of IFN-γ release. Overall, at d180, a borderline T-cell response (cutoff > 100 mIU/ml) as well as a stronger response (cutoff > 200 mIU/ml) was detectable in all the 81 experienced. Among 271 naive, 7 (2.58%) and 17 (6.27%) did not show borderline or strong responses, respectively (Table 1). The difference between median IFN-γ concentration of 254 (93.7%) naive and 81 (100%) previously experienced subjects was significant with values of 223.0 (463.0–2,272.0) mIU/ml vs. 2,499.0 (2,400.0–2,500.0) mIU/ml, respectively, (*p* < 0.0001) when IFN-γ concentration higher than 200 IU/ml was analyzed.

### Correlation Between IgG Levels and IFN-γ in Naive

**Figure 6:**
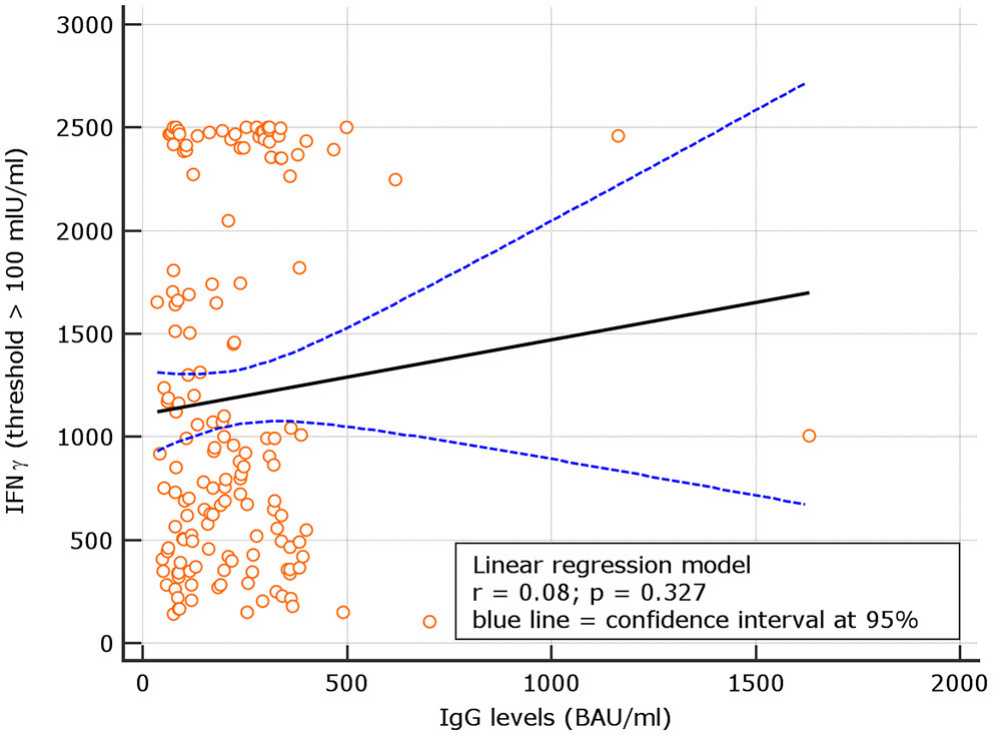
Day 180, linear regression between IgG levels and IFN-γ concentration among naive group using the IFN-γ threshold of 100 mIU/ml.

**Figure 7:**
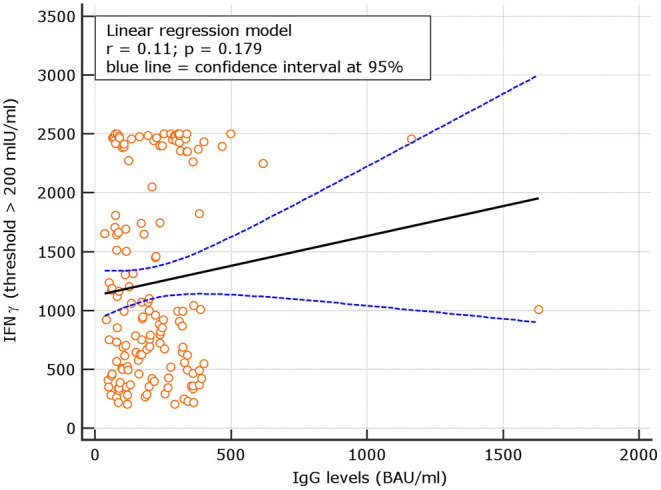
Linear regression between IgG levels and IFN-g concentration among COVID naive group using the IFN-γ threshold of 200 mIU/ml.

Levels of IgG at d180 were correlated with IFN-γ concentrations in subjects with results >100 IU/ml. A not significant correlation with *r* = 0.08, *p* = 0.344 was observed. Using a IFN-γ threshold > 200 IU/ml, a similar not significant correlation with *r* = 0.11, *p* = 0.192 was found (Figures 6, 7). At variance, when levels of IgG at d60, 90 days after the first vaccine dose (5) were correlated with IFN-γ concentrations in subjects with results >100 IU/ml, at that time point, results were statistically significant *r* = 0.28, *p* = 0.031; similar results were attained using at d90 the threshold of >200 IU/ml (additional Figures 1, 3). Thesedata support an overtime decline of humoral response but not of lymphocyte IFN-γ.

### Correlation Between Neutralizing Antibodies and IFN-γ

**Figure 8:**
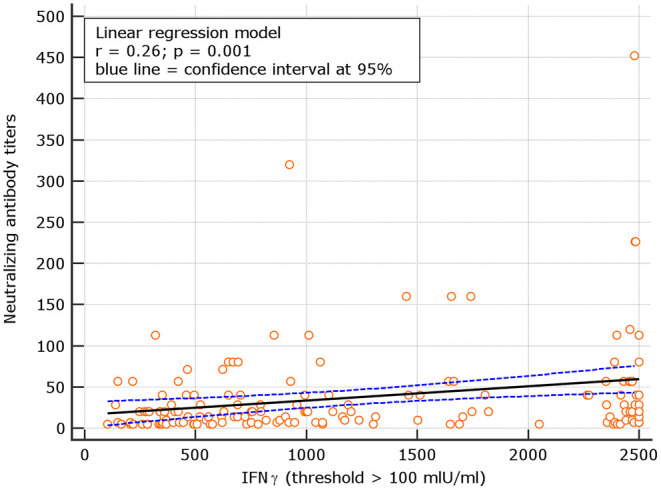
Linear regression model between neutralizing antibody titers and IFN-γ concentration in naive (with IFN-γ threshold > 100 mIU/ml).

**Figure 9:**
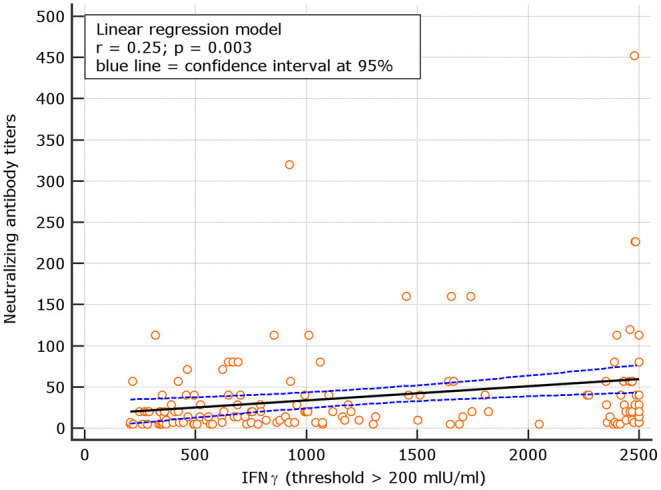
Linear regression model for correlation between neutralizing antibody dilutions and IFN-γ concentration in naive (with IFN-γ concentration > 200 mIU/ml).

**Figure 10:**
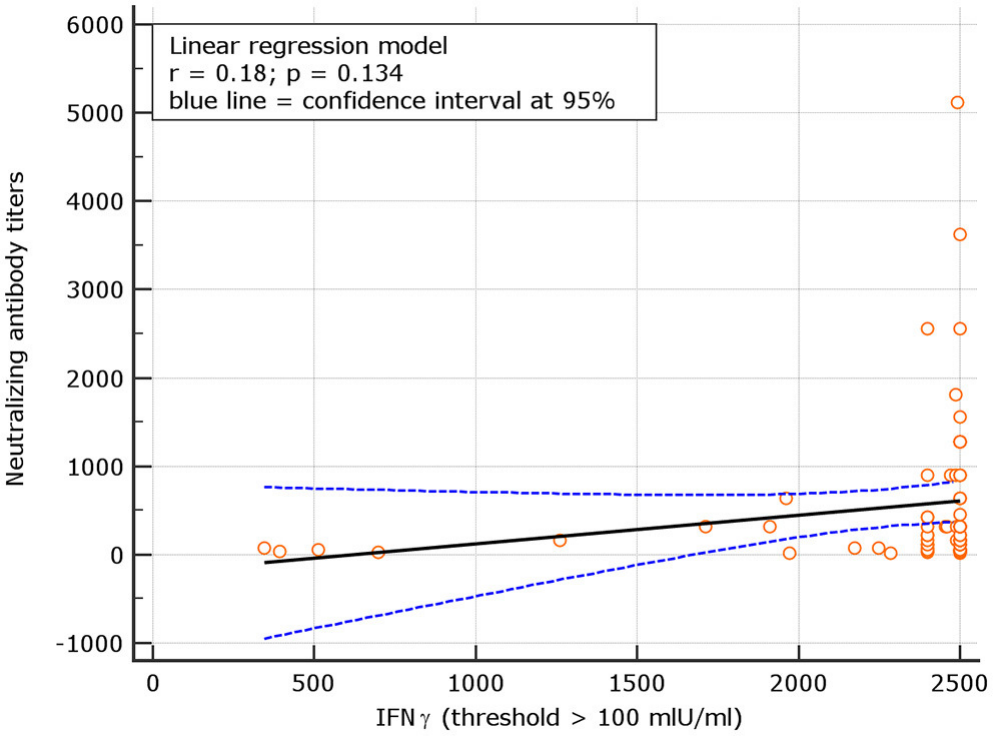
Linear regression model for correlation between neutralizing antibody titers and IFN-γ concentration in experience (similar results for IFN-γ threshold of 100 and 200 mIU/ml given the identical number of subjects above these thresholds among experienced).

An interesting correlation between neutralizing titers and IGRA levels was found for both naive and experienced. The results showed *r* = 0.26; *p* = 0.001 for naive and *r* = 0.18 *p* = 0.134, respectively (Figures 8, 9). The significance of the correlation increased for naive when the IFN-γ positive threshold of 200 was used (*r* = 0.25; *p* = 0.003) and did not change for experience given the identical number of subjects with IFN-γ concentration >100 and >200 thresholds in this group (Figure 10). The regression curves for naive (at both IFN-γ positivity thresholds) and experienced are reported in Figures 8–10.

### Breakthrough Infections

Breakthrough infections were observed in 6 cases among naive fully vaccinated subjects (2.2%). Characteristics of subjects experiencing infection are shown in Table 3. In all the cases, the infection was mild, none of the subjects required hospitalization. A persistently positive swab result was observed in almost all (mean positivity duration 4.5 ± 2.3 weeks). For 4 out of 6, a common unvaccinated index case was identified. The remaining two cases came from the same household, where one of the individuals, a healthcare worker, was exposed and exposed to the second individual within the household. Demographic, virologic, and immunologic characteristics of these subjects were compared with those of the remaining not infected naive subjects (Table 3). Our small group of subjects with breakthrough infection showed simultaneous neutralizing antibody titers below 20, binding antibody levels below 200 BAU/ml and IFN-γ <1,000. Similar results in subjects older than 58 years may be considered an alarming condition.

**Table 3:** Characteristics of patients with breakthrough infection.

Pt initials	Gender	Age	IgG level BAU/ml	IGRA titers mIU/ml	Neutralizing antibody dilution
RF	M	35	311.14	905.1	14.1
VV	F	67	172.39	750	7.1
D'AG	M	57	105.53	420	5
VA	F	57	160	360	10
RG	F	59	200	620	20
CM	M	70	80.6	100	5

## Discussion

Our study investigated the IgG and neutralizing response in naive and experienced HW previously shown to be able to mount a strong IgG response at d31 (5). At d180 after the second BioNTech/Pfizer vaccine shot, among naive, all HW had binding antibody levels higher than the assay threshold, although only 8.1% had results higher than the highest assay threshold. At variance, 1/3 of subjects had neutralizing antibodies titers below LLoQ, while titers ≥ 320 generally associated with protection, were observed in very few cases (1.2%). Converting neutralizing antibody titers in International Unit (IU/ml) by running in the same neutralization assay, the first SARS-CoV-2 WHO International Standard (NIBSC 20/136)^1^, we observed that only 32.78% of our patients had 50% protective neutralizing antibody. Our results appear in keeping with those reported in two studies from Israel, where the majority of the population was vaccinated using the BioNTech/Pfizer or Moderna vaccine. The first study on over 1,000,000 persons (596,618 vaccinated and 596,618 non-vaccinated) demonstrated high efficacy of vaccines, not only in disease prevention but also in infection transmission up to 42 days after the first vaccination (7). A second more recent study with longer follow up from the same Country, showed that 39 (2.6%) out of 1,497 fully vaccinated HW became infected during 14 weeks after their second dose of the BNT162b2 (BioNTech/Pfizer) vaccine; all the infected had lower neutralizing antibody levels than their uninfected colleagues during the peri-infection period (23). In our study, only 6 subjects (2.2%) experienced a breakthrough infection. All of them were older and had median neutralizing antibody levels lower than the median of the uninfected population. Although we are aware that our sample size is limited, our results appear in line with those reported in Israel.

The already known significant decline in BNT162b2 vaccine protection more than 120 days after the second dose, in our study, conducted in the region of Puglia with a low community incidence rate (positivity index on December 16, 2021, was 2.4%)^2^, was associated with the rate of breakthrough infections comparable to those reported by Bergwek (23) and were significantly lower than the rates reported among unvaccinated subjects^3^.

In keeping with the decreased severity of the disease in vaccinated individuals who acquire SARS-CoV-2 infection, all our patients with breakthrough infections were mild. A persistently positive swab result was observed in almost all (mean positivity duration 4.5 ± 2.3 weeks). Whether a possible further decrease in vaccine effectiveness against hospitalization after a longer interval from vaccination occurs was impossible to evaluate in our population given the mandatory administration of a third vaccine dose to the HW in Italy that started from November 22, 2021, based on the evidence that booster dose may mitigate the risk of transmission, disease, and deaths in all the age groups (24)^4^.

Reliable detection of the T-cell-mediated immune response was explored in our study by IFN-γ production. Most of the subjects showed robust IFN-γ production after S-protein stimulation of peripheral blood cells. Results below the threshold of the assay were observed in only 12 (4.6%) naive, suggesting that lack of T-cell reactivity is a rare event even after a long interval from the second vaccine shot. This evidence was also confirmed by the cytoflorimetric analysis (manuscript in preparation). Moreover, as shown by the linear regression model, higher T-cell reactivity was observed in patients with higher neutralizing antibody levels. These results are in agreement with those reported by Schiffner et al. (25, 26). Consequently, the combination of these two assays seems to provide predictive information on protective immune reactions. Nevertheless, we need to keep in mind that neutralizing titers may be impractical to assess routinely, whereas IFN-γ evaluation as an expression of lymphocyte activity may be easier to use than other more complex CD4+ and CD8+ cellular response assessment methods.

Whether the decay of serum antibody levels is a good indicator for the timing of booster administration remains to be determined. Identifying immune correlates of protection (or lack thereof) from SARS-CoV-2 is critical in predicting how the expected antibody decay will affect clinical outcomes, if and when a booster dose will be needed, and whether vaccinated persons are protected (23, 26). Surely antibody decay represents one of the initial predisposing factors to breakthrough infections. However, while cellular and humoral immunity to SARS-CoV-2 is critical to control primary infection and correlates with severity of disease, the degree of vaccine protection from breakthrough infections may be an expression of the initial immune response rather than of the decay of antibody levels, since memory cells are expected to respond to future exposures. Moreover, while correlates of protection have been developed for other infections such as influenza (27) by challenge experiments in humans (28), no study has defined correlate of protection until a recent one that focused on correlates of protection against symptomatic COVID-19 (29, 30). This study highlights that there is no single threshold value for different assays (31). In our small group of subjects who experienced a breakthrough infection, we had the opportunity to both identify a common source of infection in an unvaccinated index case and to show low median neutralizing antibody titers and higher median age.

The use of the same mRNA vaccine with a similar schedule and similar interval between vaccination and post-vaccination antibody assessment strengthen this study. Moreover, evaluating one of the longest delays between the second vaccine dose and both IgG and neutralizing antibody assessment has the advantage of using the IFN-γ spike-specific-induced T-cell immune response assay that allows simultaneous cellular responses evaluation. Finally, we had the opportunity to trace the incident breakthrough infection and to investigate its possible predictors. Limitations of our study are the relatively small sample size, the homogeneous demographic characteristics of our patients, young and healthy in the majority of cases. A further disadvantage is the relatively low prevalence of SARS-CoV-2 infection in our region as compared to others in Italy. This may prevent the exportability of our findings to the general population with different ages and co-morbidities.

In conclusion, our study shows that although the low humoral response is relatively long-lasting, high IgG levels are extremely rare in naive subjects. Only a third of subjects maintained neutralizing responses. In terms of T-cell, IFN-γ production after specific stimulation, a very limited number of subjects resulted unable to produce this cytokine over a period of 180 days after the second shot. IFN-γ testing could be used as surrogate testing for cellular immune responses. The results attained in our small group of subjects with breakthrough infection suggest that simultaneous neutralizing antibody titers below 20, binding antibody levels below 200 BAU/ml, and IFN-γ <1000 in subjects older than 58 years may be considered an alarming condition.

## Data Availability Statement

The datasets presented in this study can be found in online repositories. The names of the repository/repositories and accession number(s) can be found below: https://zenodo.org/record/5728042#.Ycy6BWnSJPw.

## Ethics Statement

The studies involving human participants were reviewed and approved by EC IST Giovanni Paolo II IRCCS, BARI at Fondazione Casa Sollievo della Sofferenza San Giovanni Rotondo. The patients/participants provided their written informed consent to participate in this study.

## Author Contributions

AMang: conceptualization, data curation, formal analysis, investigation, methodology, project administration, supervision, validation, visualization, writing – original draft, and writing – review & editing. VP: data collection and writing – original draft. GC: formal analysis and data collection. VG: investigation and writing – original draft. AMane: formal analysis, investigation, writing – original draft, and writing – review & editing. PC: formal analysis and investigation. EM: writing – review & editing. FG: visualization. AMai: validation. SA: formal analysis and investigation. All authors contributed to the article and approved the submitted version.

## Funding

This study was partially funded by Ministry of Health of Italy, Bando Ricerca COVID-19; Project Number: COVID-2020-12371619; project title: COVIDIAGNOSTIX—Health Technology Assessment in COVID serological diagnostics.

## Conflict of Interest

EM, PC, and AMane were employed by VisMederi Srl. The remaining authors declare that the research was conducted in the absence of any commercial or financial relationships that could be construed as a potential conflict of interest.

## Publisher's Note

All claims expressed in this article are solely those of the authors and do not necessarily represent those of their affiliated organizations, or those of the publisher, the editors and the reviewers. Any product that may be evaluated in this article, or claim that may be made by its manufacturer, is not guaranteed or endorsed by the publisher.

## Footnotes

^1^ https://www.nibsc.org/documents/ifu/20-136.pdf

^2^https://bari.repubblica.it/argomenti/coronavirus_puglia (accessed December 16, 2021).

^3^https://www.epicentro.iss.it/coronavirus/bollettino/Bollettino-sorveglianza-integrata-COVID-19_7-dicembre-2021.pdf (accessed December 16, 2021).

^4^https://www.salute.gov.it/portale/nuovocoronavirus/dettaglioComunicatiNuovoCoronavirus.jsp?menu=salastampa&id=5830 (accessed December 16, 2021).

## Supplementary Material

Supplementary Figure 1 Day 60 Linear regression between IgG levels and IFN-γ concentration among COVID naïve group using the IFN-γ threshold of 100 mIU/ml.

Supplementary Figure 2 Day 60 Linear regression between IgG levels and IFN-γ concentration among COVID naive group using the IFN-γ threshold of 200 mIU/ml.
