# Supplementary figures and images for: Cellular and Humoral Immune Responses and Breakthrough Infections After Two Doses of BNT162b Vaccine in Healthcare Workers (HW) 180 Days After the Second Vaccine Dose
Source: Front Public Health. 2022 Mar 31;10:847384. doi: 10.3389/fpubh.2022.847384 (PMC9008351; doi:10.3389/fpubh.2022.847384)

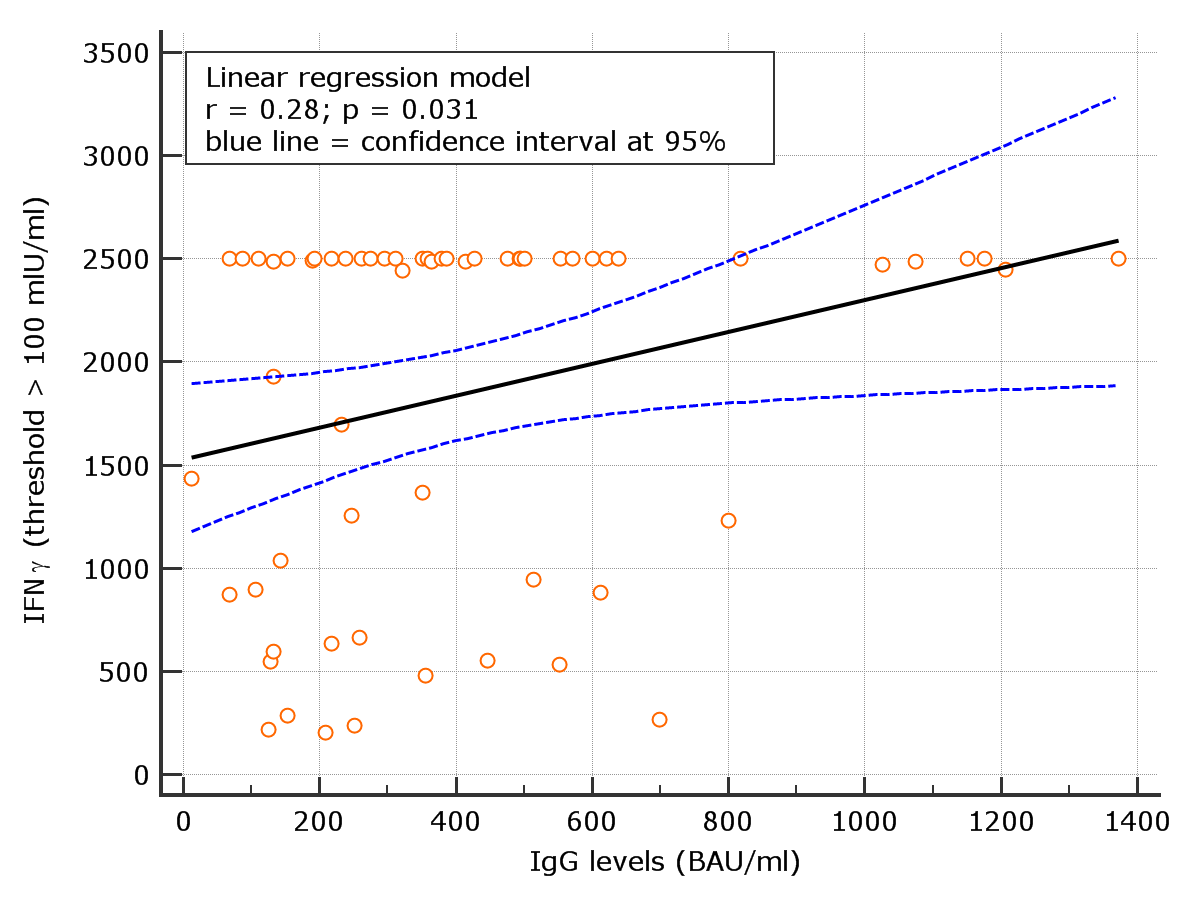

Supplement: Supplementary Figure 1 — Day 60 Linear regression between IgG levels and IFN-γ concentration among COVID naïve group using the IFN-γ threshold of 100 mIU/ml. [file Image_1.TIFF]

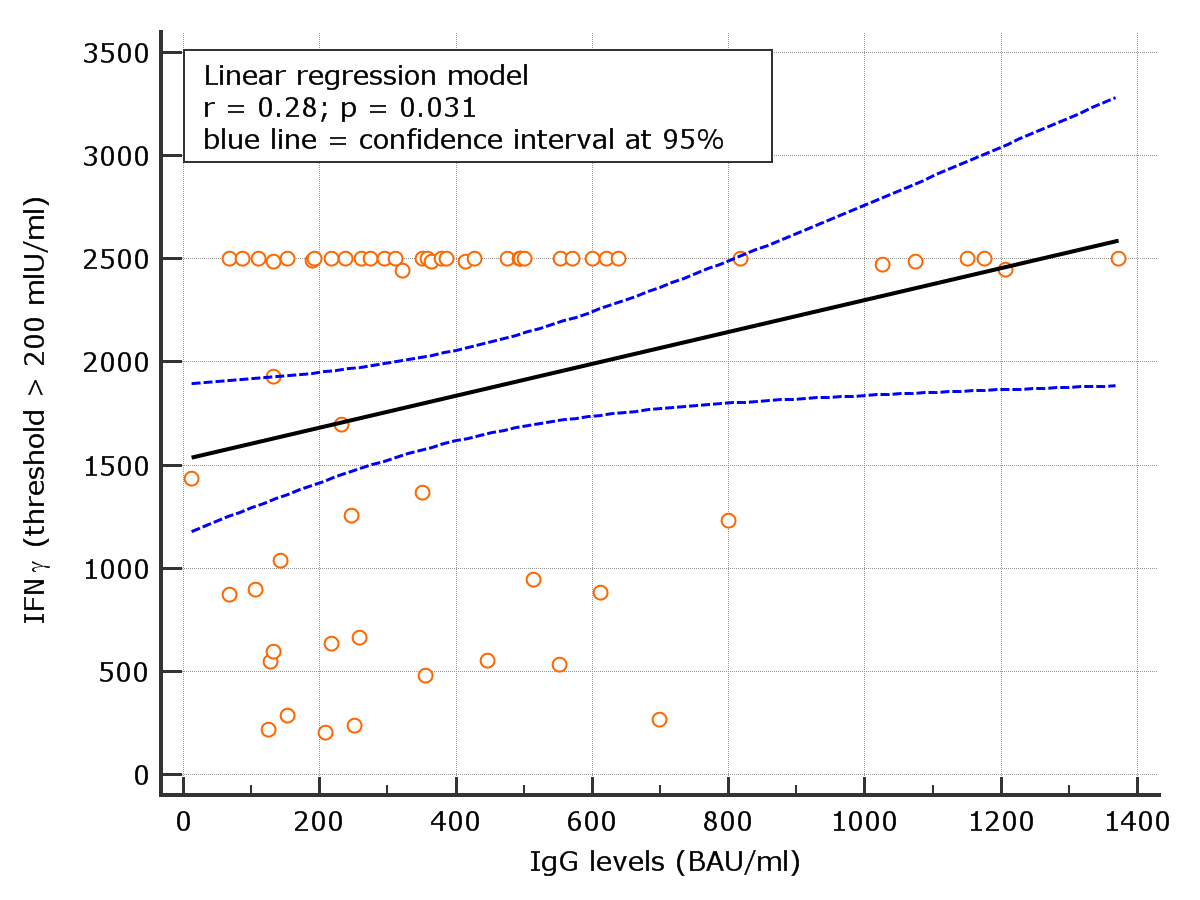

Supplement: Supplementary Figure 2 — Day 60 Linear regression between IgG levels and IFN-γ concentration among COVID naive group using the IFN-γ threshold of 200 mIU/ml. [file Image_2.TIFF]
